# β-Hydroxyisovalerylshikonin regulates macrophage polarization *via* the AMPK/Nrf2 pathway and ameliorates sepsis in mice

**DOI:** 10.1080/13880209.2022.2046111

**Published:** 2022-03-30

**Authors:** Tao Pan, Yabin Chang, Min He, Zehui He, Jun Jiang, Xinling Ren, Fang Zhang

**Affiliations:** a Translational Medicine Center, The Second Affiliated Hospital of Guangzhou Medical University, Guangzhou, Guangdong, China; b Department of Respiratory Medicine, Xijing Hospital, Air Force Medical University, Xi'an, Shaanxi, China; c Shaanxi Key Laboratory of Brain Disorders, Institute of Basic Medical Sciences, Institute of Basic and Translational Medicine, Xi'an Medical University, Xi'an, Shaanxi, China; d Carson International Cancer Center, Shenzhen University, Shenzhen, Guangdong, China; e Shenzhen University Clinical Medical Academy, Shenzhen, Guangdong, China

**Keywords:** Chinese herb extract, non‑cytotoxic dose, lipopolysaccharide, anti-inflammatory effect

## Abstract

**Context:**

The potential anti-inflammatory bioactivities of β-hydroxyisovalerylshikonin (β-HIVS) remain largely unknown.

**Objective:**

This study investigated the anti-inflammatory effects and underlying mechanisms of β-HIVS.

**Materials and methods:**

RAW 264.7 cells stimulated with LPS (100 ng/mL) for 24 h were treated with the non-cytotoxic doses of β-HIVS (0.5 or 1 μM, determined by MTT and Trypan blue staining), qRT-PCR and FCM assay were used to examine macrophage polarization transitions. Western blotting was used to evaluate the activation of the AMPK/Nrf2 pathway. *In vivo*, C57BL/6 mice were randomly divided into vehicle control, LPS (10 mg/kg), and β-HIVS (2.5 mg/kg) combined with LPS (10 mg/kg) groups, blood samples, BALF, and lung tissues of mice were subjected to ELISA, qRT-PCR, FCM, and H&E staining.

**Results:**

β-HIVS (1 μM) inhibited LPS-induced expression of M1 macrophage markers (TNF-α: 0.29-fold, IL-1β: 0.32-fold), promoted the expression of M2 macrophage markers (CD206: 3.14-fold, Arginase-1: 3.98-fold) in RAW 264.7 cells; mechanistic studies showed that β-HIVS increased the expression of nuclear Nrf2 (2.04-fold) and p-AMPK (3.65-fold) compared with LPS group (*p* < 0.05). *In vivo*, β-HIVS decreased the levels of pro-inflammatory cytokines (TNF-α: 1130.41 *vs.* 334.88 pg/mL, IL-1β: 601.89 *vs.* 258.21 pg/mL in serum; TNF-α: 893.07 *vs.* 418.21 pg/mL, IL-1β: 475.22 *vs.* 298.54 pg/mL in BALF), decreased the proportion of M1 macrophages (77.83 *vs.* 68.53%) and increased the proportion of M2 macrophages (13.55 *vs.* 19.56%) in BALF, and reduced lung tissue damage and septic mice survival (*p* < 0.05).

**Conclusions:**

These results indicate that β-HIVS may be a new potential anti-inflammatory agent.

## Introduction

Inflammation is a complex biological process involving physio-pathological functions (Feng et al. 2019). The inflammatory response is initiated by foreign invaders and/or sterile damaged tissues, and displays the characteristics of high expression of pro-inflammatory cytokines (Feng et al. 2014). Generally, inflammation can protect the body from injury or infection by endogenous and exogenous pathogens; it can also lead to serious tissue damage once excessive and unresolved inflammatory responses (Grivennikov et al. 2010; Sindrilaru et al. 2011). Sepsis can be triggered by an over-activated immune system as a defense mechanism for eliminating infectious pathogens and is the leading cause of mortality for critically ill patients admitted to the intensive care units (Dellinger et al. 2013; Wang, Huang, et al. 2018). Given that there is no effective treatment strategy until now, this condition is still a major threat to human health. Therefore, the development of new agents targeting this condition is urgently needed (Feng et al. 2019).

As an important part of innate immunity, the monocyte-macrophage system plays an important role in pathogen elimination, host defense, anti-inflammation, and tissue repair (Gordon and Martinez 2010; Sica and Mantovani 2012). Upon sensing various exogenous and endogenous stimuli in different types of tissues, macrophages can be reprogrammed into the corresponding phenotypes, including classical M1 activation and alternative M2 activation, or other states between these two extreme phenotypes (Mantovani et al. 2002, 2013). M1 and M2 macrophages exhibit significantly different chemokine profiles. In this case, M1 macrophages typically generate high levels of pro-inflammatory cytokines, including TNF-α, IL-1β, and reactive nitrogen, which promote Th1 reaction and destroy bacteria. On the contrary, M2 macrophages express anti-inflammatory cytokines that function in the immunomodulatory process, tissue repair, and tumour progression (Feng et al. 2014). A mutual conversion between M1 and M2 phenotypes can be observed to a certain extent both *in vitro* and *in vivo* (Saccani et al. 2006). In the early stage of sepsis, the macrophages engulf bacteria and produce various pro-inflammatory cytokines that elicit the innate immune responses (Benoit et al. 2008). The above macrophages are thought to display an M1-polarized phenotype. In patients suffering from severe sepsis, the circulating concentrations of M1-type cytokines were found to be positively correlated with mortality rates (Benoit et al. 2008; Feng et al. 2014). In contrast to M1-polarized macrophages, M2 counterparts can protect the body from the damage caused by severe inflammatory reactions (Wei et al. 2018). Regulation of M1/M2 macrophage phenotypes has been shown to effectively alleviate sepsis in mice (Feng et al. 2019). Thus, targeting the macrophage polarization may be a promising strategy for the treatment of severe inflammatory diseases, such as sepsis (Feng et al. 2014).

As one of the major active components of the traditional Chinese herb Zicao (the dried root of *Lithospermum erythrorhizon* Siebold & Zucc., a medicinal plant in the family Boraginaceae), β-hydroxyisovalerylshikonin (β-HIVS) was shown to suppress the proliferation of tumour cells and promote apoptosis in a variety of malignancies, such as leukaemia (Hashimoto et al. 1999; Masuda et al. 2003, 2004), lung cancer (Xu et al. 2004), prostate cancer (Liu et al. 2010), and cervical cancer (Lu et al. 2015). However, the potential anti-inflammatory bioactivities of β-HIVS and the underlying mechanisms still need to be explored. This study investigates the potential effect of β-HIVS on the regulation of macrophage activation as well as the underlying mechanisms. Here, we reported that β-HIVS inhibited LPS-induced M1 macrophage activation and promoted the polarization of M2 macrophages *via* activation of AMPK/Nrf2 signalling. In addition, β-HIVS exhibited a significant anti-inflammatory effect *in vivo* and relieved mouse sepsis induced by LPS.

## Materials and methods

### Reagents and chemicals

Chemicals and reagents were purchased as follows: β-HIVS (>98% purity) from Yuanye Biotechnology Co., Ltd. (Shanghai, China), lipopolysaccharide (LPS) and Compound C (CC) from Sigma-Aldrich (St. Louis, MO, USA), *tert*-butylhydroquinone (tBHQ) from MedChem Express (NJ, USA), 3-[4, 5- dimethylthylthiazol-2-yl]-2,5-diphenyltetrazolium bromide (MTT) from Beyotime Biotechnology (Nantong, Jiangsu, China), Trypan blue and all of the cell culture reagents from Gibco (Thermo Fisher Scientific, Inc., Waltham, MA, USA).

### Cell culture

Mouse macrophage cell line (RAW 264.7) was provided by the Cell Bank of Type Culture Collection at the Chinese Academy of Sciences (Shanghai, China). The cells were routinely grown in DMEM culture medium containing 10% FBS and antibiotics as described previously (Zhang et al. 2014).

### Isolation and culture of murine bone marrow-derived macrophages (BMDMs)

As described previously (Zhang et al. 2019), bone marrow was flushed from the femurs of C57BL/6J mice (Shanghai Laboratory Animal Company, Shanghai, China). After collection and centrifugation, the dissociated cells from bone marrow were incubated with Red Blood Cell Lysis Buffer (Beyotime, Shanghai, China) on ice for 10 min, centrifuged, and then resuspended in DMEM with 10% FBS plus 10 ng/mL of macrophage colony-stimulating factor (PeproTech, NJ, USA). Subsequently, the cells were seeded in 6-well culture plates at a concentration of 1 × 10^6^ cells/mL (2 mL/well), followed by incubation at 37 °C and 5% CO_2_. After 7 days in culture, the remaining adherent BMDMs were used for subsequent assays. The procedures for the care and use of the animals were approved by the experimental animal ethics committee of Xi'an Medical University (XYLS2020154), and all relevant institutional and governmental regulations concerning the ethical use of animals were followed.

### Assessment of cytotoxicity

MTT assay was conducted to assess the viability of cells. The cells were first grown in 96-well culture plates at 37 °C for 24 h. Afterwards, 5 mg/mL MTT was applied to each well (20 μL/well), followed by incubation for another 4 h. Following incubation, the supernatants in each well were removed gently, and DMSO (150 μL/well) was used to dissolve formazan crystals (Zhang et al. 2014). A ELX 800 microplate reader (Bio-Tek Instruments, Winooski, VT, USA) was utilized to measure OD values at 570 nm.

The percentage of dead cells was determined by Trypan blue staining (Wright et al. 2012; Li et al. 2015). After treatment, the cells were collected, centrifuged, and stained with 0.4% Trypan blue. The proportion of dead cells (stained) to the total number of cells (stained and unstained) was determined by counting with a haemocytometer.

### ELISA assay

Quantikine ELISA kit (Beyotime Biotechnology, Shanghai, China) was used to measure the extracellular levels of TNF-α in the culture medium according to the manufacturer’s instructions. Briefly, the medium was collected by centrifugation at 500 *g* for 10 min. The culture supernatant was added into anti‑TNF‑α antibody‑coated wells and incubated for 2 h at room temperature. Then, the corresponding biotinylated antibody was added to each well and incubated for 1 h at room temperature. Horseradish peroxidase (HRP)-streptavidin was added and incubated for 20 min at room temperature in the dark. Then, TMB substrate was added into each well and incubated for another 20 min at room temperature in the dark. Stop solution was added and gently mixed for 2 min at room temperature in the dark. The absorbance values at 450 nm were detected using a microplate reader (ELX 800; BioTek Instruments, Inc.), and the concentration of TNF‑α was calculated by referring to the standard curve (Pan et al. 2020; Zhang et al. 2020).

### Flow cytometry assay

The apoptosis of RAW 264.7 cells was assessed using a Dead Cell Annexin‑V‑FITC Propidium iodide (PI) apoptosis detection kit (Invitrogen; Thermo Fisher Scientific, Inc.) according to the manufacturer's protocol. The rate of apoptotic cells was evaluated using a standard EPICS Elite flow cytometer (Beckman Coulter, Inc.) as previously described (Zhang et al. 2020), and the data were analyzed with CXP Analysis Software version 1.0 (Beckman Coulter, Inc.).

To distinguish M1 and M2 macrophage polarization transitions after β-HIVS treatment, CD86 was chosen to mark the M1 phenotype and CD206 for the M2 phenotype (Ding et al. 2021). RAW 264.7 cells were harvested by centrifugation after the indicated treatment, and the supernatant was discarded. Subsequently, the cells were washed with PBS at 4 °C and centrifuged twice at 100 *g* for 5 min. The cell density was adjusted to 1 × 10^6^ cells/mL (Ding et al. 2021). Thereafter, the cells were suspended in 300 µL binding buffer and incubated with anti-CD86-PE-Cy5 (BioLegend, San Diego, CA, USA), isotype control-PE-Cy5 (BioLegend), anti-CD206-APC (BioLegend), or isotype control-APC (BioLegend) for 30 min at 4 °C. After incubation, about 30,000 cells were selected from each sample and suspended in 300 µL of PBS. The cells were subjected to flow cytometric analysis using a NovoCyte Flow Cytometer (ACEA Biosciences, Inc.), and data analysis was conducted with FlowJo software (Tree Star, Ashland, OR, USA).

### Quantitative real-time PCR

Total RNA isolation and qRT-PCR assay were conducted as described previously (Pan et al. 2017). Briefly, total RNA was isolated by the Invitrogen Trizol Reagents. AMV reverse transcriptase (Promega, Madison, WI, USA) was used to synthesize the corresponding cDNA. Quantitative real-time PCR was conducted in triplicate using SYBR Premix Ex Taq (Takara Bio Inc., Kusatsu, Japan) and ABI Prism 7500 real-time PCR instrument. After normalization with GAPDH, the relative quantification of target genes was performed using the 2^−ΔΔCt^ method (Livak and Schmittgen 2001). The primer sequences for each gene are presented in Table 1.

**Table 1.** PCR primers for the indicated genes.

Gene	Forward	Reverse
TNF-α	5′-CATCTTCTCAAAATTCGAGTGAC-3′	5′-TGGGAGTAGACAAGGTACAACCC-3′
IL-1β	5′-TGGAAAAGCGGTTTGTCTTC-3′	5′-TACCAGTTGGGGAACTCTGC-3′
CD206	5′-CTTCGGGCCTTTGGAATAAT-3′	5′-TAGAAGAGCCCTTGGGTTGA-3′
Arginase-1	5′-GTGAAGAACCCACGGTCTGT-3′	5′-GCCAGAGATGCTTCCAACTG-3′
HO-1	5′-GAGATAGAGCGCAACAAGCAG-3′	5′-CTTGACCTCAGGTGTCATCTC-3′
NQO-1	5′-GCCTGAGCCCAGATATTGTG-3′	5′-GGAAAGGACCGTTGTCGT-3′
GAPDH	5′-GGCCTTCCGTGTTCCTAC-3′	5′-TGTCATCATATCTGGCAGGTT-3′

### Nrf2 knockdown

Nrf2 siRNA (5′-CCGAATTACAGTGTCTTAA-3′) and scrambled control siRNA (5′-GCCAGACTAACATGACTTCGA-3′) were designed and synthesized by Hanbio Biotechnology (Shanghai, China). Growing cells at 50% confluence were transfected with 24 nM of Nrf2 siRNA or the same concentration of the control siRNA in 12-well culture plates. RNAi^MAX^ was premixed with siRNAs in OPTI medium according to the manufacturer’s instructions (Invitrogen), and the mixture was then applied to the cultured cells. After 48 h of transfection, the FBS-containing DMEM medium was substituted with the OPTI medium (Wang, Xu, et al. 2018), and the transfected cells were subsequently incubated with β‑HIVS and/or LPS for the indicated time periods.

### Western blot analysis

The nuclear protein and total protein were isolated using a nuclear protein extraction kit (Beyotime Biotechnology) and RIPA buffer (Thermo Fisher Scientific, Inc.), respectively. The protein concentrations were assessed using a BCA protein assay kit (Beyotime Biotechnology) according to the manufacturer’s instructions. Equal amounts of protein samples (30 μg protein per lane) were separated by 10% SDS-PAGE and transferred onto PVDF membranes (Servicebio, Wuhan, Hubei, China). The membrane was blocked in 5% non-fat skim milk for 1 h at room temperature and then incubated with primary antibodies overnight at 4 °C. The following antibodies were used: anti-AMPKα (1:1000 dilution; Cell Signalling Technology, Beverly, MA, USA), anti-p-AMPKα (1:1000 dilution; Cell Signalling Technology), anti-Nrf2 (1:1000 dilution; Cell Signalling Technology), anti-HO-1 (1:1000 dilution; Cell Signalling Technology), anti-NQO-1 (1:1000 dilution; Abcam, Cambridge, UK), anti-histone H3 (1:1000 dilution; Beyotime Biotechnology), and anti-β-actin (1:1000 dilution; Beyotime Biotechnology). On the next day, the blot was washed, followed by incubation with anti-rabbit or anti-mouse horseradish-peroxidase-conjugated secondary antibodies (Proteintech, Wuhan, Hubei, China) at 1:5000 dilution for 1 h. The target proteins were detected by ECL reagents (Millipore, Billerica, MA, USA). Densitometry-based semi-quantification of protein levels was performed using ImageJ version 1.46r (NIH, Bethesda, MD, USA) (Schneider et al. 2012; Wallmeyer et al. 2017).

### LPS-induced sepsis

LPS-induced mouse sepsis was established as described previously (Feng et al. 2014; Feng et al. 2019). After acclimating to the new rearing environment for 7 days, female C57BL/6 mice (Vital River Laboratory Animal Technology Company, Beijing, China) aged 6–8 weeks, weighing 18–22 g, were randomly allocated into three groups (*n* = 10 per group). Among the three groups, one was intraperitoneally injected with β-HIVS (2.5 mg/kg) for three consecutive days, while the other two groups received the same treatment with vehicles (10% DMSO + 40% PEG300 + 5% Tween-80 + 45% saline, all from MedChemExpress, Monmouth Junction, NJ, USA). After 3 days of treatment, the mice received an intraperitoneal injection of 10 mg/kg LPS or normal saline at 2 h following β-HIVS administration. Six hours later, blood samples, bronchoalveolar lavage fluid (BALF), and lung tissue were collected from each mouse and subjected to ELISA assay, qRT-PCR, flow cytometry assay, or hematoxylin-eosin (H&E) staining, respectively. The procedures for the care and use of the animals were approved by the experimental animal ethics committee of Xi'an Medical University (XYLS2020154), and all relevant institutional and governmental regulations concerning the ethical use of animals were followed.

### BALF collection and detection

Following LPS administration for 6 h, the mice were sacrificed and subjected to BALF collection using 0.5 mL PBS three times (Feng et al. 2019). The recovery rate of BALF reached ∼90%. Then, the BALF samples were centrifuged at 1500 *g* for 10 min at 4 °C, and the supernatants were collected. The ELISA kits (Beyotime Biotechnology, Shanghai, China) were used to measure the levels of cytokines in the samples as described previously (Zhang et al. 2020).

Before flow cytometric analysis, the collected BALF was plated in 12-well plates, and the adhered alveolar macrophages were repeatedly washed, harvested, and resuspended in staining buffer (MultiSciences, Hangzhou, Zhejiang, China) (Wang, Xu, et al. 2018). After treatment with FcR Blocking Reagent (Miltenyi, Auburn, CA, USA), the cells were stained with anti-F4/80-FITC (BioLegend), fixed and permeabilized with fixation/permeabilization buffer (Thermo Fisher Scientific, Inc.), and then stained with anti-CD86-PE-Cy5 (BioLegend) or anti-CD206-APC (BioLegend) antibody. The data were acquired using a NovoCyte Flow Cytometer (ACEA Biosciences, Inc.), and the FlowJo software was used for data analysis.

### Histological analysis

Mouse left lungs were removed at 6 h following LPS administration. Histopathological analysis was conducted on the mice treated as above without collecting BALF samples. After fixation in 10% neutral buffered formalin for 48 h, the lung tissues were dehydrated, embedded in paraffin, and cut into sections (5 μm thick). Afterwards, the sections were subjected to H&E staining for assessing inflammatory cell infiltration and lung tissue damage (Lv et al. 2017). Histological examination of the stained sections was performed using a light microscope.

### Statistical analysis

All experiments were performed at least three times independently. The data are presented as mean ± *SD*. Statistical analyses were conducted using SPSS Statistics ver. 16.0 software (SPSS Inc., Chicago, IL, USA). The differences among the groups were statistically analyzed by one-way analysis of variance (ANOVA) and Bonferroni’s *post-hoc* test. The log-rank test of Kaplan–Meier survival curves was employed to compare intergroup survival rates. *p* < 0.05 was considered statistically significant.

## Results

### β-HIVS inhibits macrophage M1 polarization and promotes M2 activation in RAW 264.7 cells

The chemical structure of β-HIVS is depicted in Figure 1(A). The MTT assay, Trypan blue staining, and flow cytometry assay revealed that β-HIVS induced significant cytotoxicity and lead to RAW 264.7 cell apoptosis at the concentration of 1.5 μM, while 0.5 or 1.0 μM of β-HIVS showed no marked cytotoxicity (Figures 1(B–D)). The ELISA results showed that the non‑cytotoxic doses of β-HIVS (0.5 and 1.0 μM) markedly suppressed LPS-elicited TNF-α secretion in RAW 264.7 cells (Figure 1(E)). The real-time PCR assay further demonstrated that the non‑cytotoxic doses of β-HIVS profoundly decreased LPS-induced expression of M1 macrophage phenotype-related genes (TNF-α and IL-1β), while rescuing the decreased expression of M2 macrophage marker genes (CD206 and Arginase-1) induced by LPS (Figure 1(F)). The maximal effect was achieved at the dose of 1 μM. Based on these results, β-HIVS at the dose of 1.0 μM was selected for subsequent experiments. The flow cytometry assay clearly showed that β-HIVS suppressed M1 polarization and facilitated M2 activation in RAW 264.7 cells stimulated with LPS (Figure 1(G)). Moreover, treatment with β-HIVS upon or after LPS stimulation also led to a significant inhibition in the expression of LPS-elicited M1 macrophage marker genes as well as increased expression of M2 macrophage marker genes in RAW 264.7 cells (Figures 1(H,I)).

Figure 1.β-HIVS inhibits the polarization of M1 macrophages and promotes M2 macrophage activation in RAW 264.7 cells. (A) Chemical structure of β-HIVS. (B–D) RAW 264.7 cells were treated with β-HIVS at various concentrations (0, 0.5, 1.0, and 1.5 μM) for 24 h. Then, MTT assay (B), Trypan blue staining (C) and flow cytometry assay (D) were used to detect the cell survival rates, the percentages of dead cells and apoptosis, respectively. *p < 0.05 compared with the control group (c). (E,F) RAW 264.7 cells were pre-treated with β-HIVS at the indicated doses for 1 h, followed by incubation with 100 ng/mL LPS for another 24 h. After that, ELISA assay (E) and real-time PCR assay (F) were employed to measure TNF-α contents in cell medium, as well as the mRNA levels of M1 marker genes (TNF-α and IL-1β) and M2 marker genes (CD206 and Arginase-1) in RAW 264.7 cells, respectively. (G) RAW 264.7 cells were pre-treated with 1 μM β-HIVS for 1 h, followed by incubation with 100 ng/mL LPS for another 24 h. After treatment, the percentage of CD86^+^ M1 macrophages or CD206^+^ M2 macrophages was detected by flow cytometry. (H,I) The cells were either simultaneously incubated with 1 μM β-HIVS and 100 ng/mL LPS for 24 h (H), or treated with 100 ng/mL LPS for 1 h, followed by 1 μM β-HIVS for an additional 24 h (I). After treatment, the mRNA levels of M1 and M2 marker genes were detected by real-time PCR assay. ^#^*p* < 0.05 and *p < 0.05 *vs.* the control and LPS alone groups, respectively.

**Figure:**
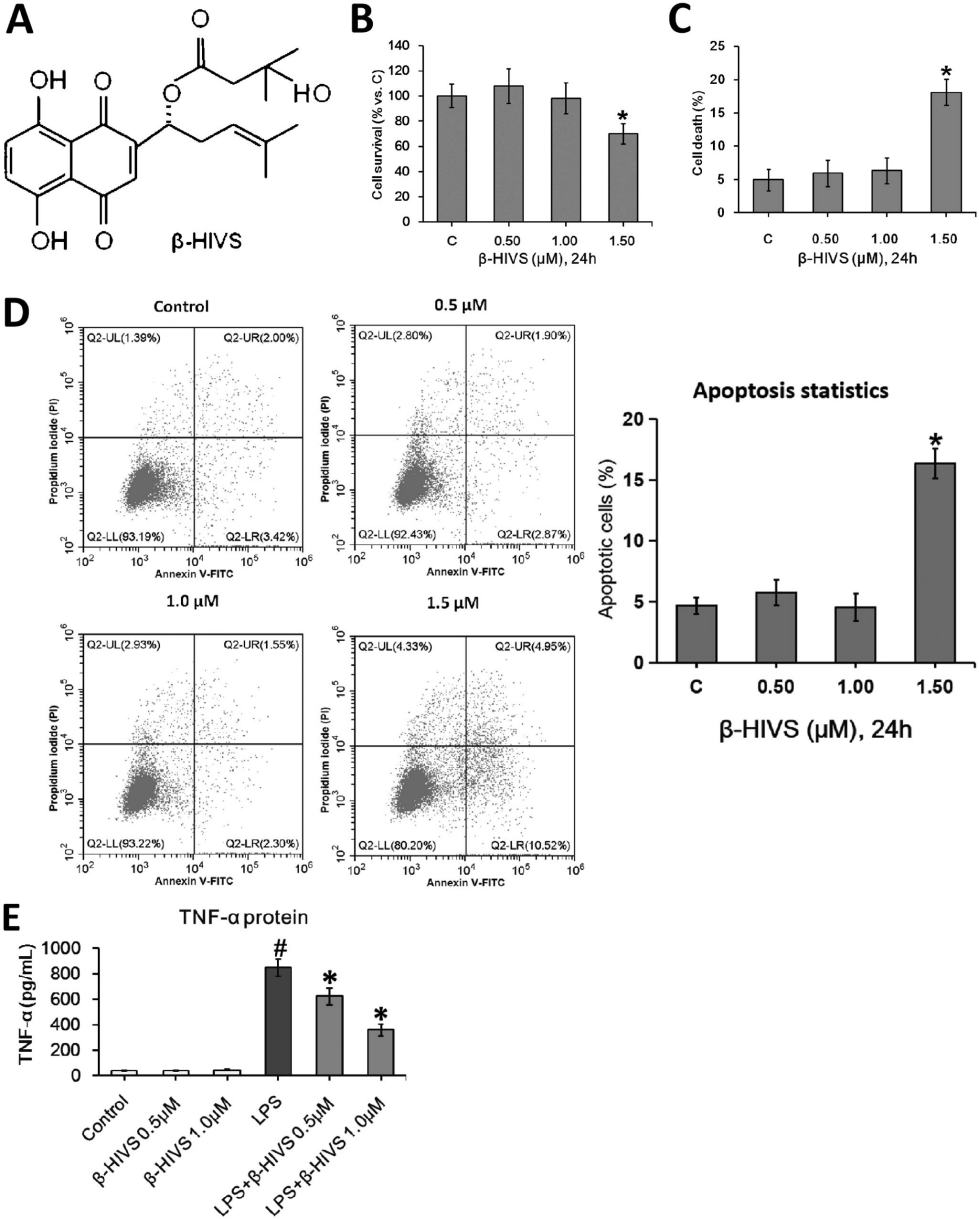

**Figure:**
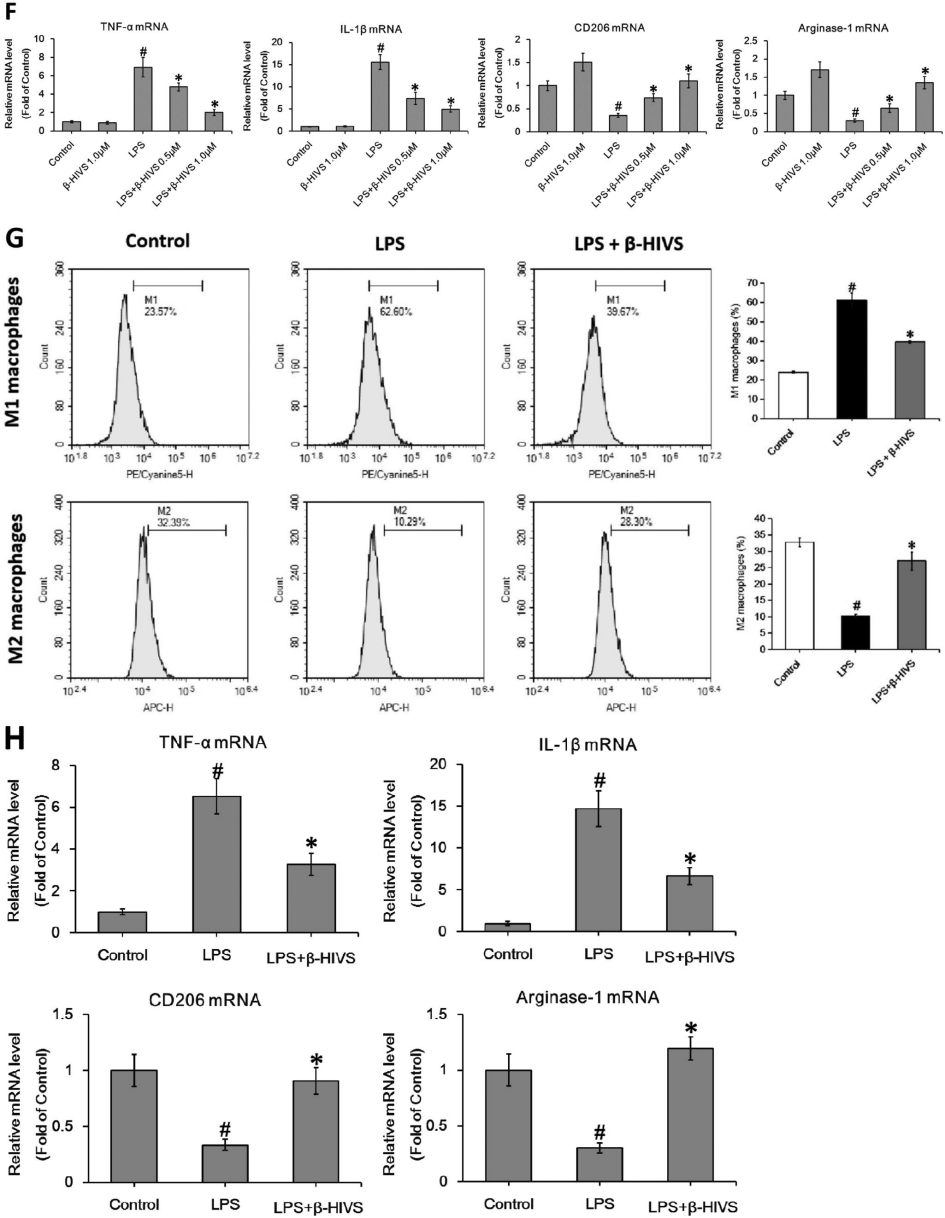

**Figure:**
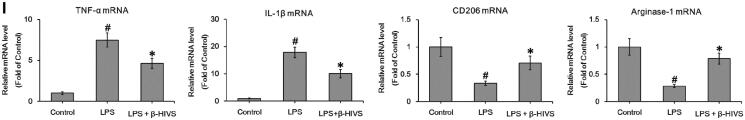

### β-HIVS regulates macrophage polarization via activation of Nrf2 signalling

Accumulating evidence indicates that Nrf2 signalling pathway plays an important role in anti-inflammatory responses (Saha et al. 2020). In this study, β-HIVS promoted the nuclear accumulation of Nrf2 in RAW 264.7 cells treated with or without LPS (Figures 2(A,B)), while increasing the expression of HO-1 and NQO-1, the downstream targets of Nrf2 in cells (Figure 2(C)). Furthermore, as shown in Figures 2(D–G), knocking down of Nrf2 significantly attenuated the regulatory effect of β-HIVS on macrophage polarization in RAW 264.7 cells. In addition, the classic Nrf2 activator tBHQ could also inhibit LPS-induced expression of M1 marker genes and enhance the expression of M2 marker genes in RAW 264.7 cells (Figure 2(H)). Collectively, these results suggest that Nrf2 signalling pathway is critically involved in macrophage polarization, and β-HIVS regulates macrophage polarization *via* Nrf2 signalling.

Figure 2.β-HIVS regulates macrophage polarization through the activation of Nrf2 signalling. (A) RAW 264.7 cells were incubated with 1 μM β-HIVS for different time periods. Then, Western blot analysis was used to detect the levels of nuclear Nrf2. *p < 0.05 compared with the ‘0 h’ group. (B,C) The cells were incubated with 1 μM β-HIVS for 1 h, followed by 100 ng/mL LPS for another 3 h (B) or 24 h (C). Then, Western blotting was performed to detect the levels of nuclear Nrf2, HO-1, and NQO-1. ^#^*p* < 0.05 and *p < 0.05 *vs.* the control and LPS alone groups, respectively. (D) The cells were incubated with Nrf2 siRNA or control siRNA (NC) for 24 h, and then subjected to the detection of relative mRNA levels of Nrf2 using real-time PCR assay. *p < 0.05 *vs.* the NC siRNA group. (E–G) After 48 h of Nrf2 siRNA transfection, the cells were incubated with 1 μM β-HIV for 1 h, followed by 100 ng/mL LPS for another 3 h (E) or 24 h (F,G). Western blot assay (E) was used to examine the levels of nuclear Nrf2, real-time PCR assay (F) and flow cytometry assay (G) were used to examine the changes in macrophage polarization, respectively. *p < 0.05 *vs.* the NC siRNA group. (H) The cells were incubated with 1 μM β-HIVS or 100 μM tBHQ for 1 h, followed by 100 ng/mL LPS for an additional 24 h. Then, real-time PCR assay was conducted to measure the mRNA levels of M1 and M2 marker genes. ^#^*p* < 0.05 and *p < 0.05 *vs.* the control and LPS alone groups, respectively.

**Figure:**
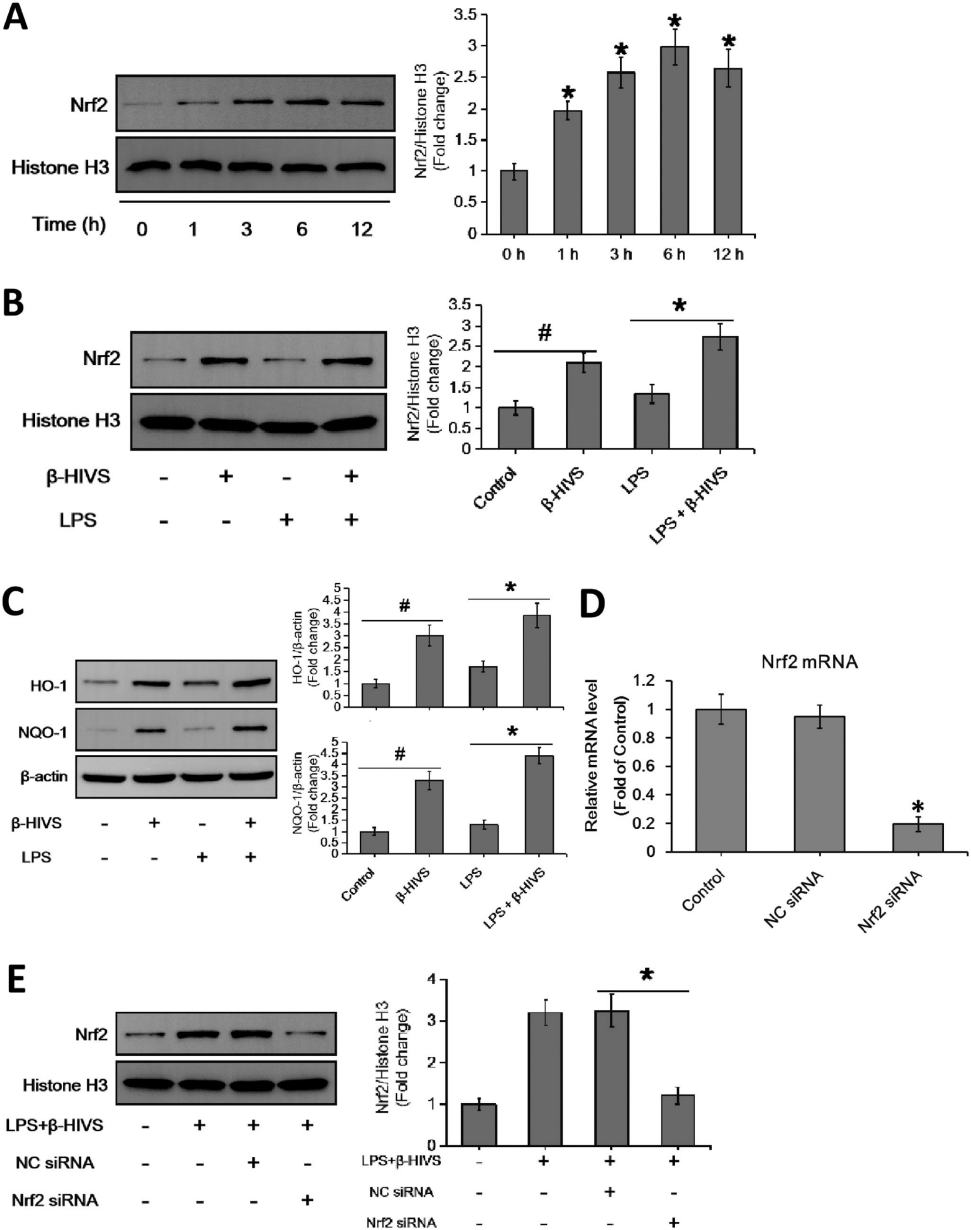

**Figure:**
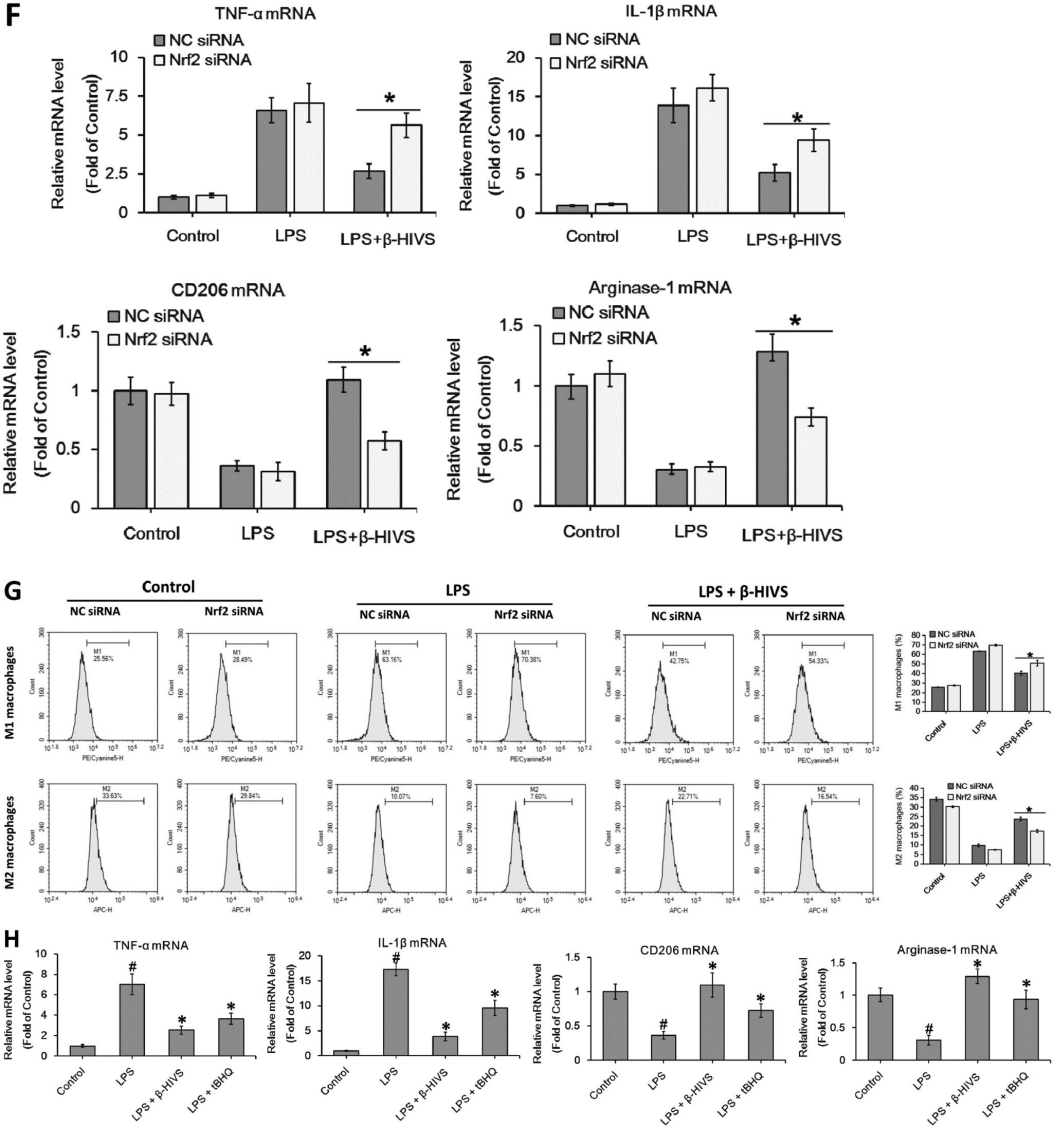

### Identification of AMPK as an upstream mediator of Nrf2 activation during β-HIVS-regulated macrophage polarization

It has been shown that AMPK is closely related to Nrf2 in the regulation of inflammatory responses (Lv et al. 2017; Qiu et al. 2018; Zhou et al. 2019). Here, we found that β-HIVS markedly promoted AMPK activation in RAW 264.7 cells treated with or without LPS (Figures 3(A,B)), pre-treatment with the AMPK inhibitor compound C could block the activation of AMPK pathway induced by β-HIVS (Figure 3(B)). Notably, compound C not only attenuated the β-HIVS-induced expression of nuclear Nrf2 as well as its targets HO-1 and NQO-1 (Figures 3(C,D)), but also reversed the regulatory effect of β-HIVS on macrophage polarization in RAW 264.7 cells (Figures 3(E,F)). These results suggest that AMPK mediates Nrf2 signalling activation in RAW 264.7 cells during β-HIVS-regulated macrophage polarization.

Figure 3.AMPK acts as an upstream mediator of Nrf2 activation during β-HIVS-regulated macrophage polarization. (A) RAW 264.7 cells were incubated with 1 μM β-HIVS for different time periods, and Western blot analysis was conducted to detect the levels of p-AMPKα (Thr-172) and AMPKα. *p < 0.05 compared with the ‘0 h’ group. (B–F) The macrophages were incubated with 1 μM β-HIVS alone or combined with CC (Compound C, 10 μM, 30 min earlier) for 1 h, followed by 100 ng/mL LPS for another 3 h (B,C) or 24 h (D–F). After treatment, Western blot analysis was used to detect the levels of p-AMPKα/AMPKα (B), nuclear Nrf2 (C), HO-1, and NQO-1 (D). ^$^*p* < 0.05, ^#^*p* < 0.05, ^△^*p* < 0.05, and *p < 0.05 *vs.* the control, β-HIVS alone, LPS alone, and LPS + β-HIVS groups, respectively. Real-time PCR assay (E) and flow cytometry assay (F) were performed to examine the changes in macrophage polarization, respectively. ^$^*p* < 0.05, ^#^*p* < 0.05, and *p < 0.05 *vs.* the control, LPS alone, and LPS + β-HIVS groups, respectively.

**Figure:**
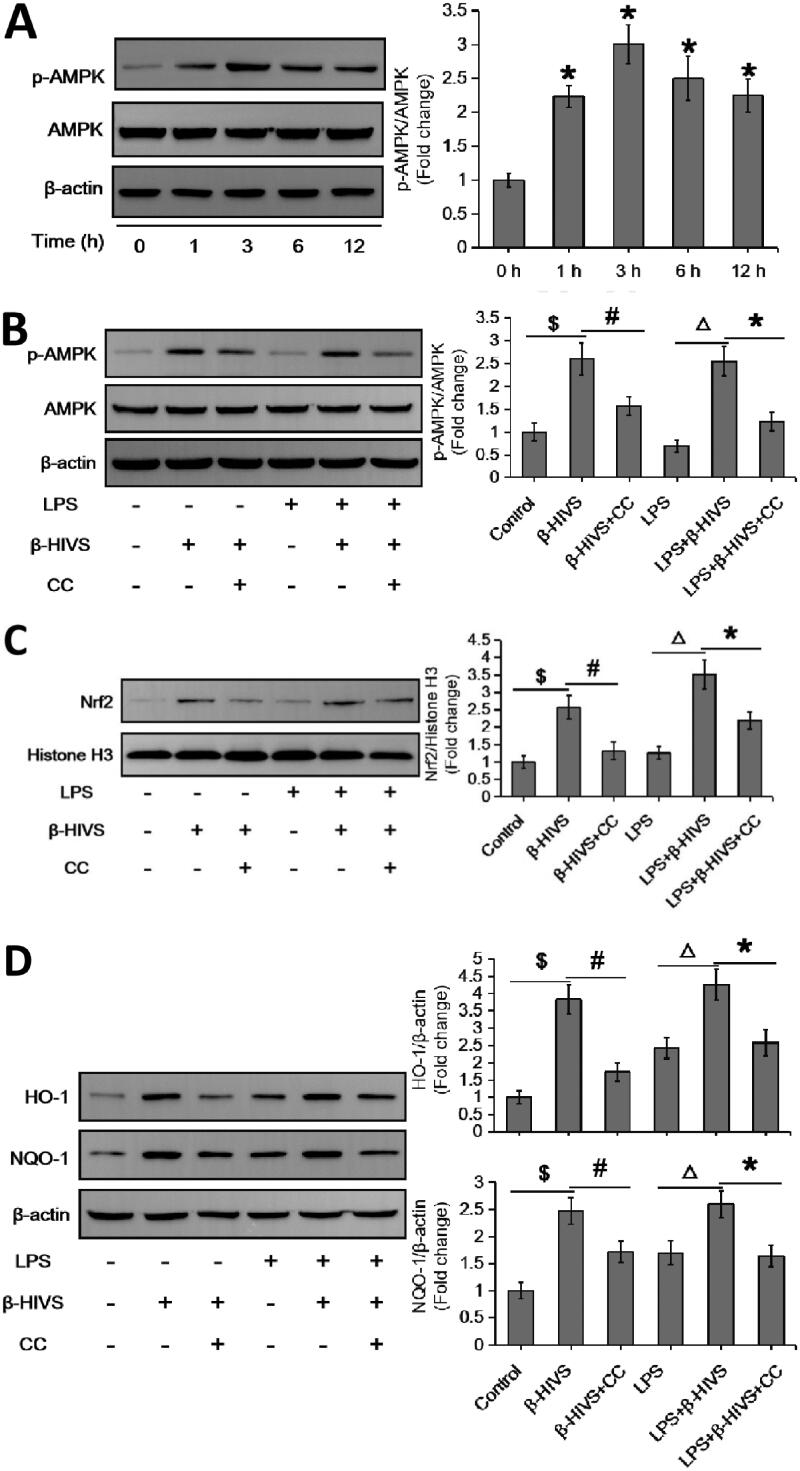

**Figure:**
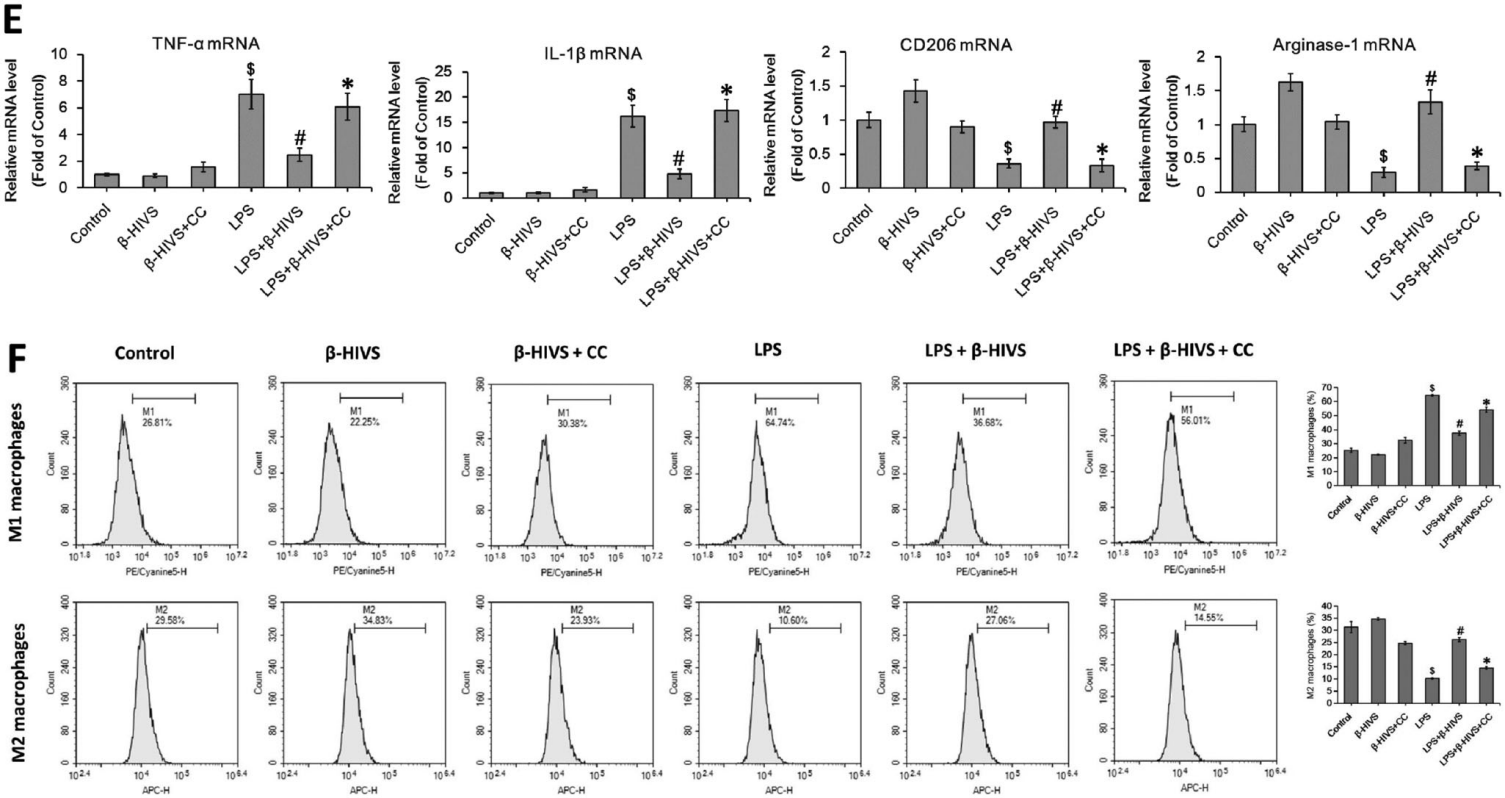

### β-HIVS also regulates macrophage polarization in primary murine BMDMs via AMPK/Nrf2 pathway

**Figure 4.**
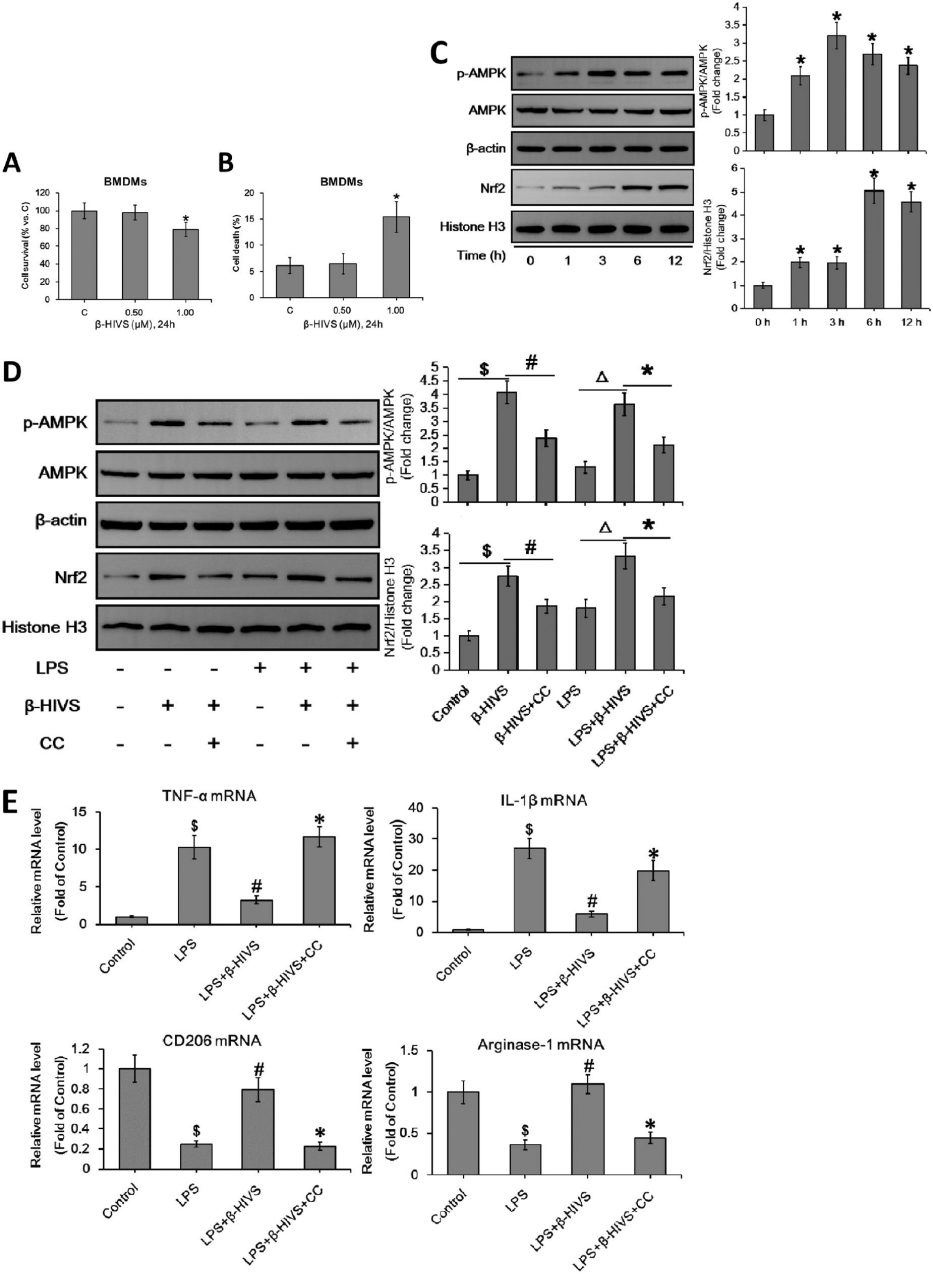
β-HIVS regulates macrophage polarization in primary murine BMDMs *via* AMPK/Nrf2 pathway. (A,B) Primary murine BMDMs were incubated with β-HIVS at the indicated doses for 24 h. Then, MTT assay (A) and Trypan blue staining (B) were employed to assess the survival and death rates of BMDMs, respectively. *p < 0.05 compared with the control group. (C) The BMDMs were treated with 0.5 μM β-HIVS for different time periods, and the levels of p-AMPKα/AMPKα and nuclear Nrf2 were measured by Western blot analysis. *p < 0.05 compared with the ‘0 h’ group. (D,E) The BMDMs were pre-treated with 0.5 μM β-HIVS alone or combined with CC (10 μM, 30 min earlier) for 1 h, followed by 100 ng/mL LPS for an additional 3 h (D) or 24 h (E). After treatment, Western blot analysis was conducted to detect the levels of p-AMPKα/AMPKα and nuclear Nrf2 (D). ^$^*p* < 0.05, ^#^*p* < 0.05, ^△^*p* < 0.05, and *p < 0.05 *vs.* the control, β-HIVS alone, LPS alone, and LPS + β-HIVS groups, respectively. In the meantime, the mRNA levels of M1 and M2 marker genes were detected using real-time PCR assay (E). ^$^*p* < 0.05, ^#^*p* < 0.05, and *p < 0.05 *vs.* the control, LPS alone, and LPS + β-HIVS groups, respectively.

Furthermore, the regulatory effects of β-HIVS on macrophage polarization in primary murine BMDMs were investigated. MTT assay and Trypan blue staining revealed that 0.5 μM was the non-cytotoxic dose of β-HIVS (Figures 4(A,B)). As shown in Figures 4(C,D), β-HIVS (0.5 μM) profoundly increased the expression levels of p-AMPKα and nuclear Nrf2 in primary murine BMDMs treated with or without LPS. Moreover, β-HIVS markedly downregulated the LPS-elicited expression of M1 marker genes, while it reversed the decreased expression of M2 marker genes induced by LPS in macrophages (Figure 4(E)). Notably, pre-treatment with compound C significantly attenuated the effects of β-HIVS as described above (Figures 4(D,E)). Collectively, these data demonstrate that β-HIVS also regulates the polarization of macrophages in primary murine BMDMs *via* AMPK/Nrf2 pathway.

### β-HIVS ameliorates LPS-induced sepsis in mice

**Figure 5.**
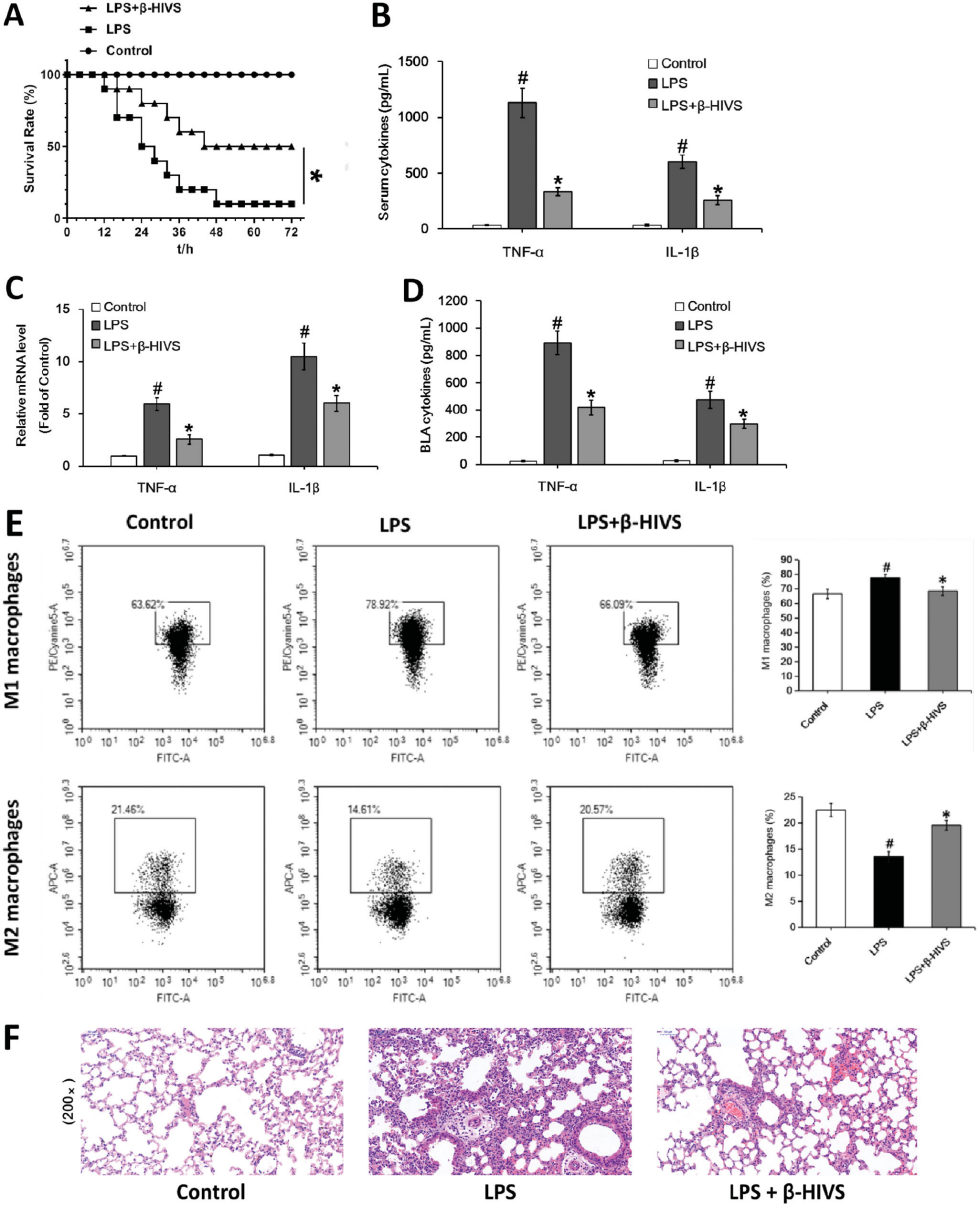
β-HIVS ameliorates LPS-induced mouse sepsis. (A) Survival rates of mice. *p < 0.05 *vs.* the LPS alone group. (B) The levels of TNF-α and IL-1β in serum. (C) The mRNA expression of TNF-α and IL-1β detected in lung tissues. (D) The levels of TNF-α and IL-1β detected in BALF. (E) The percentage of F4/80^+^ CD86^+^ M1 alveolar macrophages and F4/80^+^ CD206^+^ M2 alveolar macrophages in BALF was analyzed by flow cytometry. (F) The lung tissue sections with H&E staining. Original magnification, ×200. ^#^*p* < 0.05 and *p < 0.05 *vs.* the control and LPS alone groups, respectively.

Finally, we examined the anti-inflammatory activity of β-HIVS in LPS-induced septic mice. As displayed in Figure 5(A), treatment with β-HIVS led to a significant increase in the survival rate of septic mice. Meanwhile, β-HIVS markedly inhibited the serum levels of pro-inflammatory cytokines TNF-α and IL-1β (Figure 5(B)), indicating the inhibitory effect of β-HIVS on systemic inflammatory response in mice. Next, the possible role of β-HIVS in protecting the lung tissue was determined. As shown in Figures 5(C,D), β-HIVS treatment caused a significant inhibition in the mRNA expression of TNF-α and IL-1β in lung tissues, as well as a decrease in the secretion levels of these two cytokines in BALF. Furthermore, β-HIVS treatment decreased the proportion of M1 macrophages and increased the proportion of M2 macrophages in BALF (Figure 5(E)). Histopathological analysis revealed that treatment with β-HIVS significantly improved the infiltration of inflammatory cells as well as lung tissue damage in septic mice (Figure 5(F)). Altogether, these data show that β-HIVS ameliorates LPS-induced mouse sepsis, which is indicative of a good anti-inflammatory effect of this compound *in vivo*.

## Discussion

Multiple transcriptional factors have been shown to regulate the dynamic balance between M1 and M2 macrophages during polarization. A recent study showed that the activation of transcription factor Nrf2 could inhibit the polarization of M1 macrophages and promote the activation of M2 macrophages, thereby preventing the progression of acute respiratory distress syndrome through NF-κB inhibition (Wei et al. 2018). Moreover, Nrf2 could induce the expression of PPARγ, which in turn leads to the macrophage polarization of the M2 phenotype and protects the lung from oxidative damage (Cho et al. 2010; Luo et al. 2017). In addition, Nrf2 was found to regulate the expression of antioxidant response elements and cytoprotective genes, thereby modulating the innate immune response and protecting the body from tissue damage caused by severe inflammatory responses (Wei et al. 2018). In this study, we observed that β-HIVS, a naphthoquinone component extracted from the traditional Chinese herb Zicao, stimulated the activation of Nrf2 pathway, suppressed the polarization of M1 macrophages, and facilitated M2 macrophage activation. Further investigation demonstrated that knocking down of Nrf2 significantly attenuated the regulatory effect of β-HIVS on macrophage polarization. These results suggest that β-HIVS regulates macrophage polarization presumably through Nrf2 signalling activation.

Increasing evidence has suggested that the AMPK pathway is important for regulating inflammatory responses (Guo et al. 2014). It has been reported that AMPK activation significantly reduces the production of pro-inflammatory mediators as well as tissue injury during sepsis (Zhang et al. 2017). The activated AMPK pathway was found to enhance the polarization of macrophages towards M2 phenotypes, thereby inhibiting inflammatory response (Wang, Huang, et al. 2018; Wang, Xu, et al. 2018). Besides, multiple studies identified a close correlation of AMPK with Nrf2 in the regulation of inflammatory responses (Lv et al. 2017; Qiu et al. 2018; Zhou et al. 2019). As an upstream regulatory pathway (Mo et al. 2014), AMPK signalling could activate the Nrf2 pathway by inhibiting Nrf2 nuclear export and promoting its nuclear accumulation (Joo et al. 2016). Here, we showed that β-HIVS could promote AMPK activation, and treatment with the AMPK inhibitor not only diminished the role of β-HIVS in Nrf2 pathway activation but also reversed the regulatory effects of β-HIVS on macrophage polarization. Thus, these findings identified AMPK as an upstream mediator of Nrf2 during β-HIVS-regulated macrophage polarization. As the upstream kinase of AMPK, LKB1, or CaMKKβ can activate AMPK under different conditions. It has been reported that several anti-inflammatory compounds, including metformin, hydrogen sulphide, and 5-aminoimidazole-4-carboxamide riboside (AICAR), can activate AMPK *via* LKB1 or CaMKKβ (Wang, Xu, et al. 2018). However, whether or not AMPK is activated by β-HIVS through these two kinases remains to be studied further.

In the final part of this study, a mouse model of LPS-induced sepsis was used to examine the *in vivo* anti-inflammatory activity of β-HIVS (Feng et al. 2014, 2019). We observed that treatment with β-HIVS led to a significant increase in the survival rate of mice and a dramatic inhibition in the levels of serum pro-inflammatory cytokines. This suggests that β-HIVS treatment can alleviate the severity of systemic inflammation. The lungs have been demonstrated to be the most vulnerable organs to sepsis, and protecting the lungs from inflammation has been considered a promising strategy for the treatment of sepsis (Wang, Xu, et al. 2018). Here, we found that β-HIVS remarkably downregulated the levels of pro-inflammatory cytokines both in lung tissue and BALF. Furthermore, β-HIVS treatment decreased the proportion of M1 macrophages and increased the proportion of M2 macrophages in BALF. Histopathological analysis showed that β-HIVS markedly ameliorated inflammatory cell infiltration and lung tissue damage. All these data indicate that β-HIVS could ameliorate LPS-induced sepsis in mouse, suggesting that this compound has a good anti-inflammatory potential *in vivo*.

Nevertheless, the strong and non-selective cytotoxicity of Zicao extracts, including β-HIVS, severely limits their clinical application (Wang et al. 2012). A number of studies have reported that β-HIVS has good anti-tumour effects (Takai et al. 2010; Lu et al. 2015; Dilshara et al. 2018), which actually make use of its strong cytotoxicity. However, this cytotoxicity is also present in normal cell lines or primary cultured normal cells (Wang et al. 2012). In the present study, the non‑cytotoxic dose of β-HIVS was determined and further selected to explore its anti-inflammatory activity and the underlying mechanisms. Unlike most of the existing studies on β-HIVS, our findings of the non-cytotoxic of β-HIVS may be more easily applicable to clinical practice.

## Conclusions

This study demonstrated for the first time that β-HIVS suppressed macrophage M1 polarization and promoted M2 polarization *via* activation of the AMPK/Nrf2 pathway. Macrophage is an attractive therapeutic target. The important role of macrophage polarization in the development of diseases has attracted considerable attention in recent years. Previous studies have shown that macrophage polarization is closely linked to autoimmune diseases, rheumatoid arthritis, and obesity (Wynn et al. 2013; Ma et al. 2019), and play a vital role in the progression of sepsis (Fu et al. 2020). The results of this study imply that β-HIVS may have therapeutic potential for the treatment of these diseases, especially sepsis, and its non‑cytotoxic dose is highly favourable to applied to clinical practice.
